# Patient-Reported Outcomes During Immunotherapy for Metastatic Melanoma: Mixed Methods Study of Patients’ and Clinicians’ Experiences

**DOI:** 10.2196/14896

**Published:** 2020-04-09

**Authors:** Lærke K Tolstrup, Helle Pappot, Lars Bastholt, Ann-Dorthe Zwisler, Karin B Dieperink

**Affiliations:** 1 Department of Clinical Research University of Southern Denmark Odense Denmark; 2 Department of Oncology Odense University Hospital Odense Denmark; 3 Department of Oncology Copenhagen University Hospital Copenhagen Denmark; 4 The Danish Knowledge Centre for Rehabilitation and Palliative Care Nyborg Denmark

**Keywords:** side effects, adverse events, patient-reported outcomes, PRO-CTCAE, melanoma, eHealth, immunotherapy, patient satisfaction, CPIs, interviews

## Abstract

**Background:**

The benefits of electronic patient reported outcomes (PRO) questionnaires have been demonstrated in many settings, including in hospitals and patient homes. However, it remains to be investigated how melanoma patients and their treating clinicians experience the electronic self-reporting of side effects and the derived communication.

**Objective:**

The primary objective of this study was to examine patients’ and clinicians’ experiences with an eHealth intervention for weekly monitoring of side effects during treatment with immunotherapy.

**Methods:**

An eHealth intervention based on questions from the PRO-Common Terminology Criteria for Adverse Events (CTCAE) library was used and tested in a randomized clinical trial with patients receiving immunotherapy for malignant melanoma and clinicians at a university hospital in Denmark. On a weekly basis, patients reported their symptoms from home during the treatment via a provided tablet. The electronic patient reports were available to clinicians in the outpatient clinic. A mixed methods approach was applied to investigate the patients’ and clinicians’ experiences with the intervention. Data from patient experiences were collected in a short survey, the Patient Feedback Form. Moreover, a subset of the patients participating in the survey was interviewed about their experience. Furthermore, one focus group interview with clinicians was carried out to elucidate their views.

**Results:**

A total of 57 patients completed the Patient Feedback Form, and 14 patients were interviewed. The focus group interview included 5 clinicians. Overall, patients and clinicians were satisfied with the tool. They believed it enhanced patients’ awareness of side effects and increased their feeling of involvement. The patients reported that it was easy to fill out the questionnaire and that it made sense to do so. However, a minority of the patients expressed in the interviews that they did not believe that the health care professionals had seen their reports when they came to the clinic, and that the reporting did not lead to increased contact with the department.

**Conclusions:**

Overall, satisfaction with the eHealth intervention was high among patients and their treating clinicians. The tool was easy to use and contributed to greater symptom awareness and patient involvement. Thus, in terms of patient and clinician satisfaction with the tool, it makes sense to continue using the tool beyond the project period.

**Trial Registration:**

ClinicalTrials.gov NCT03073031; https://tinyurl.com/tjx3gtu

## Introduction

Underreporting of symptoms by clinicians in connection with cancer therapy, particularly chemotherapy and radiotherapy, is well established [1-4]. However, over the last few decades, new therapies have been developed and various kinds of immunotherapies now play an important role in fighting cancer [5]. In particular, immunotherapy has significantly improved survival in patients with melanoma [6]. However, the side effects that patients experience when treated with immunotherapy can be severe and unpredictable, and they differ immensely from the side effects experienced by patients who receive chemotherapy [5]. Furthermore, untreated toxicities may progress and become potentially life threatening [7]. Thus, toxicity monitoring may advantageously be optimized to meet the need for early detection of symptoms. Studies have demonstrated the value in using patient-reported outcomes (PROs) to detect and monitor symptoms, and to improve communication in routine care [8], and their implementation has been encouraged [9,10]. Moreover, increased inclusion of patients in their treatment has become a priority in many health care settings worldwide [11]. Similarly, there has been an increasing awareness within the Danish health care system of patients not being sufficiently involved with their treatment and care [12], despite the fact that the Danish regions recommend planning treatment *with* the patient rather than *for* the patient [13].

The use of electronic PRO questions (ePROs) to monitor symptoms has proven to be feasible in connection with scheduled consultations (ie, in the waiting area in various oncology settings) [14], and recent evidence suggests that the method is also useful at home (ie, via a link to a webpage) [15]. Studies also demonstrate that including cancer patients in the reporting of symptoms may increase their quality of life [16], and that the general acceptability of routine data collection is high [8]. With regard to immunotherapy, previous studies have examined the quality of life during treatment [17,18], but no study has examined whether patient reporting of side effects also results in improved toxicity monitoring. Therefore, we designed a randomized controlled trial (RCT), PROMelanoma (ClinicalTrials.gov NCT03073031), with the primary aim of investigating whether the severity and frequency of severe side effects can be reduced by including the patients in the reporting of symptoms on a frequent basis. Enrollment for this study has just completed.

An exploratory endpoint of PROMelanoma was to examine whether our setup of including an eHealth intervention on symptom management is implementable in clinical practice and makes the patients feel more involved in their treatment and care. Patient and clinician satisfaction with various eHealth interventions has been measured in other studies within an oncology setting to support clinical decision making and improve patient self-management [15,19]. However, many outcome measures are not sufficiently tested in clinical practice, which is imperative before implementation. To ensure the success of PRO interventions, it is vital that they are approved by the patients [20]. Thus, there is a need for more precise measures [21] that fit the patient population under investigation [22] to make sure that the PRO intervention is feasible and easy for the patient to adopt. In this regard, studies that elucidate the usefulness of a given PRO from the perspectives of patients and their treating clinicians must be carried out. To our knowledge, no study has explored how melanoma patients treated with immunotherapy experience the electronic self-reporting of symptoms using an eHealth intervention specifically designed for this patient group [23], which makes this type of study highly relevant.

However, there is no recipe for measuring the patient experience, and measurement is not routinely conducted in a standardized manner [24]. Thus, the patient experience can be captured in different ways. To acquire a broad perspective on the topic, a mixed methods approach may be most suitable. For example, a short survey can help provide feedback about the general trends, whereas in-depth interviews may provide a more detailed understanding of both the patient and clinician perspective [25]. Similarly, Hudak et al [26] suggested that it is preferable to combine a standardized quantitative measure with a qualitative method when measuring patient satisfaction. Girgis et al [15] used a similar method when they evaluated the feasibility and acceptability of real-time reporting in a cancer population.

Thus, the primary objective of this study was to examine, using both qualitative and quantitative data, patients’ and clinicians’ experiences with an eHealth intervention to monitor the side effects during treatment with immunotherapy in routine clinical practice.

## Methods

### Overall Design

**Figure 1:**
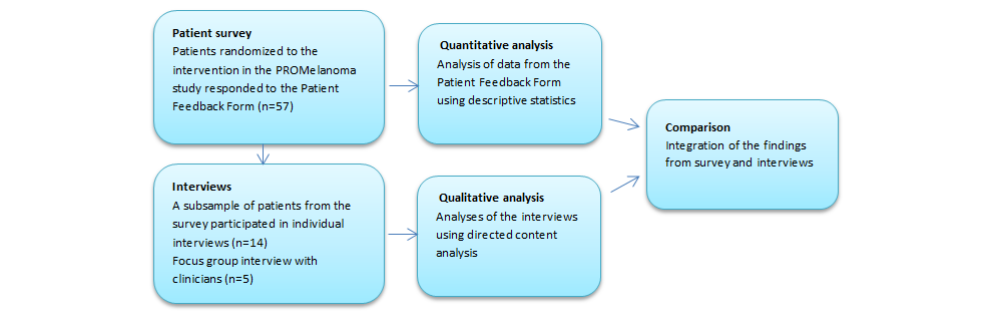
Overview of the mixed methods study design, including a survey, individual interviews, and one focus group interview.

A mixed methods approach was employed to gain deeper insight into the feasibility of the PRO intervention for melanoma patients and their treating clinicians. For quantitative assessment, a questionnaire to measure patient satisfaction, the Patient Feedback Form [19,27], was provided to patients who experienced the PROMelanoma eHealth intervention. In addition to the questionnaire, qualitative interviews with a subsample of these patients and one focus group interview with clinicians were conducted using a deductive approach [28] to evaluate the intervention. The Consolidated Criteria for Reporting Qualitative Research (COREQ) Checklist [29] was applied to ensure that all important aspects were included. A convergent design was selected [30], in which the survey data and interview data were collected in parallel over the same period of time (February 2017 to March 2019). Data were analyzed separately and compared to determine similarities and differences. Using the triangulation technique, cross verification of data from the interviews and survey was achieved (Figure 1).

### Setting

The survey and interviews took place at the Department of Oncology, Odense University Hospital, Denmark. The patients completed the Patient Feedback Form when they came to the outpatient clinic to receive their treatment for metastatic melanoma. The interviews also took place in the outpatient clinic in a separate room.

### The eHealth Intervention

**Figure 2:**
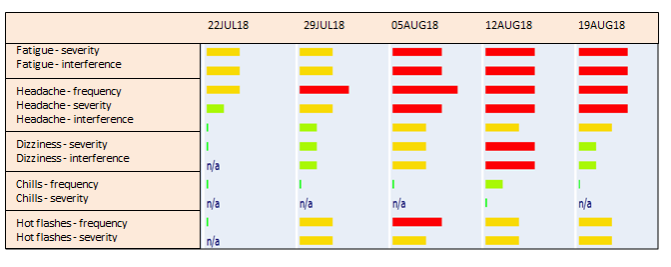
Example of part of a patient report available to clinicians.

Common Terminology Criteria for Adverse Events (CTCAE), developed by the National Cancer Institute (NCI) for patient self-reporting [31], was chosen as the PRO tool (PRO-CTCAE), since the grading scale [32] is well known within the oncology field [33] and is used by oncologists worldwide. Through a careful selection process, the relevant items were selected from the PRO-CTCAE library [34]. The software platform AmbuFlex [35] was used, which was specifically developed for ePROs. The patients received a tablet with a SIM card to ensure internet access. The reporting took place on the tablet, at home once a week, which is the preferred recall period for PRO-CTCAE items [36], and continued for 24 weeks to ensure that the majority of symptoms could be detected. The patients did not receive a weekly reminder in the form of a text message or telephone call, but they were asked to choose a fixed weekday for reporting their symptoms when they were first introduced to the intervention so that reporting would be easier to remember. If the patients experienced a symptom, an alert would tell them to contact the department. The alert function was triggered for side effects that could potentially become severe. Accordingly, side effects such as alopecia or fatigue did not trigger an alert. As soon as the patients responded to the questionnaire, the report was visible to the health care professionals at the hospital. However, clinicians did not receive a notification when an alert was triggered by a patient report; rather, it was left up to the patients to react to the alert. The clinicians only logged onto the electronic system to see the patient’s report after the patient came to the outpatient clinic. A bar attached to each symptom appeared green, yellow, or red depending on the severity of the symptom reported (Figure 2).

### Patients

Patients were eligible for the qualitative part of the study if they had been enrolled in the RCT PROMelanoma. The inclusion criteria were melanoma patients, >18 years old, randomized to the intervention in PROMelanoma, and had received at least one cycle of immunotherapy. Exclusion criteria were not able or willing to comply with the study procedure (eg, fill out the electronic questionnaire) or if they did not speak Danish.

### Survey

All patients in the PROMelanoma intervention group of the trial were asked to fill out the Patient Feedback Form between January 2017 and April 2019, which addressed patient satisfaction relating to the eHealth intervention. The Patient Feedback Form was developed by Basch et al [27] to measure patient satisfaction with the online self-reporting of toxicity symptoms, and was subsequently adapted by Snyder et al [19] who also used it to measure patient satisfaction with PRO interventions. Thus, it is an established tool to measure quantitative feedback and was considered to be appropriate for evaluating the usefulness and acceptability of our eHealth intervention. The adapted version consists of 13 items [19]. Respondents evaluate their level of agreement or disagreement on a scale with four options. Some representative questions included were: “Was it easy to use?,” “Did the questions make sense?,” and “Were the patient reports included in the patient-clinician consultation?” To apply the questionnaire for evaluating the eHealth intervention, we translated it into Danish and validated it in a Danish setting according to existing guidelines, including psychometric testing [37]. The patients had carried out the weekly PROMelanoma reporting at least three times and had the opportunity to discuss their report with a physician at least once before filling out the Feedback Form. Data were analyzed using descriptive statistics when enrollment in the PROMelanoma study closed in April 2019, and 70 patients had been enrolled in the intervention group.

### Interviews With Patients

Patients enrolled in the PROMelanoma study were contacted over the phone by the project manager and informed about this study between November 2017 and June 2018. The patients provided verbal consent and signed the written consent form in connection with the interview. We decided to use a convenience sample at the same time, taking into account the patients’ gender and age to ensure that the group was representative. The patients already had several visits scheduled in the outpatient clinic; therefore, the interviews were planned to take place on days when they were already at the hospital so as to not burden them further. If the patients were accompanied by relatives, the relatives were invited to participate in the interview. A semistructured interview guide was prepared based on the research questions, in collaboration with an expert. The interviews were carried out by the same interviewer (LT) who also carried out audio recording and transcription. The interviewer had already talked to the majority of the patients during the inclusion screening for the PROMelanoma study, but had no contact otherwise. Given that we had some knowledge about the research area in question (ie, the interviewer had worked with this patient group for more than 10 years), there were four major categories that we wished to explore: the usefulness of the eHealth solution, the questionnaire, physician-patient communication, and involvement of relatives. Thus, a directed content analysis as suggested by Hsieh and Shannon [38] was applied, using a deductive approach [39]. The fact that the level of interpretive complexity was expected to be relatively low further contributed to our choice of content analysis as the preferred method [40]. Any text that could not be categorized within the initial categories would be given a new code during the analysis [38]. Recruitment continued until data saturation was reached.

### Focus Group Interview With Clinicians

A focus group interview was chosen as the preferred method for clinicians, because the number of physicians and nurses caring for these patients was limited to a selected group, which made a questionnaire pointless. For the same reason, only one interview was conducted. The physicians and nurses who had the most experience with the intervention were chosen for the interviews. One author (KD) carried out the interview, who is a qualified researcher experienced in conducting focus group interviews. The interview was conducted in a semistructured manner [41]. Data were generated through group interaction about the specific topic predetermined by the research group. The purpose of the focus group was to explore the perspectives of the clinicians regarding the implementation and acceptability of the eHealth intervention in routine cancer care. The interview was transcribed by LT. The same content analysis approach was applied in relation to the group interview as described above [38].

## Results

### Survey

All patients who were randomized to the intervention arm in the PROMelanoma study (N=70, median age 65 years, 33 men and 37 women) were expected to evaluate the eHealth intervention by filling out the Patient Feedback Form (Table 1). However, 2 patients who had been randomized to the intervention group did not wish to proceed with the electronic reporting, and 2 patients were hospitalized due to side effects and never received the second series. For 9 patients, the melanoma progressed quickly and their conditions deteriorated, making it unethical to ask them to participate. Thus, a total of 57 patients evaluated the intervention. As summarized in Table 1, none of the patients found the eHealth intervention to be too time-consuming (item 1). In fact, one patient thought that it was too short. Similarly, almost all of the patients found the frequency with which the eHealth intervention was administered (item 2) to be just right. The general satisfaction was high. The lowest satisfaction ratings were obtained for items 8, 9, and 10, dealing with inclusion of the patient response in treatment and care. Overall, the majority of patients agreed/strongly agreed that the doctor used the information for their care, that the questionnaire improved the quality of care (item 9), and that the questionnaire improved communication with the doctor (item 10). The proportion of patients who responded “strongly agree,” “agree,” or “just right” was over 90% for 8 of the 13 questions. All of the patients (100%) recommended filling out the questionnaire to other patients and stated that they would like to continue responding to the questionnaire (items 12 and 13).

**Table 1:** Evaluation of the eHealth intervention PROMelanoma in a Danish study with patients with melanoma cancer (N=57).

Patient feedback form item	Response, n (%)		
	Category 1	Category 2	Category 3
1. Time it took to complete	1 (2)^a^	54 (94)^b^ and 0 (0)^c^	2 (4)^d^
2. Number of times completing	1 (2)^e^	54 (94)^b^ and 1 (2)^f^	1 (2) ^d^
3. Easy to complete	56 (98)^g^	1 (2)^h^	0 (0)^d^
4. Completing was useful	55 (96)^g^	2 (4)^h^	0 (0)^d^
5. Easy to understand	53 (93)^g^	4 (7)^h^	0 (0)^d^
6. Easier to remember symptoms and side effects	52 (91)^g^	4 (7)^h^	1 (2)^d^
7. Improved discussions with clinician	51 (89)^g^	4 (7)^h^	2 (4)^d^
8. Clinician used information for my care	48 (84)^g^	6 (11)^h^	3 (5)^d^
9. The quality of care improved because of the questionnaire	43 (75)^g^	8 (14)^h^	6 (11)^d^
10. Communication with clinician improved	45 (78)^g^	6 (11)^h^	6 (11)^d^
11. Made me more in control of care	50 (87)^g^	6 (11)^h^	1 (2)^d^
12. Recommend to other patients	57 (100)^g^	0 (0)^h^	0 (0)^d^
13. Would like to continue responding	57 (100)^g^	0 (0)^h^	0 (0)^d^

^a^Too short.

^b^Just right.

^c^Too long.

^d^Missing.

^e^Not often enough.

^f^Too often.

^g^Strongly agree/agree.

^h^Disagree/strongly disagree.

### Patient Interviews

#### General Characteristics of the Interviews

In addition to filling out the Patient Feedback Form, 16 of the patients were invited to participate in an in-depth interview about their experience. One patient declined and one patient who had agreed to participate was hospitalized due to deteriorating disease before the interview was conducted. Thus, 14 interviews were conducted. The median age of the patients was 67 years (range 41-79 years), including 6 men and 8 women. Apart from one patient who had only self-reported their symptoms 3 times, the patients had reported between 6 and 24 times (weeks), and the majority (10, 71%) had reported more than 15 times. Relatives were present during 10 of the interviews. The interviews lasted on average 20 minutes (range 9-33 minutes). Nine interviews lasted for more than 20 minutes. A total of 280 minutes of interview data were available for analysis. The three themes identified from the transcripts aligned with three of the predetermined categories. However, a fourth theme (involvement of relatives) did not become a theme when the final analysis was carried out.

#### Usefulness of the eHealth Solution

Overall, the patients reported that accessing and filling out the eHealth questionnaire was easy. Only two patients were not used to electronic devices upon entering the study. One of them stated “I’m pleasantly surprised. I think it is really easy to deal with” (man, 79 years old), and his wife (73 years old) added, “I did not think he could do it because he is a clown when it comes to computers…” Some of the patients, particularly the elderly, had a hard time using the touchscreen function with their fingers because they either pressed too hard or for too long. However, when they were given a touchscreen pen, which is more accurate than the fingertip, they did not have any problems. Only one patient could not do it and asked his wife to do the reporting following his instructions. Almost all of the patients experienced a request to update the operating system of the tablet while using it, but they were able to close the message easily and continued their reporting. Otherwise, there were only minor technical challenges, and the patients were very compliant and contacted the department in case of any technical problems. The majority of patients were pleased with the tablet. Only a few patients would have preferred a link instead of having to take home the tablet. As mentioned above, it was not possible to send a text message reminding the patient to fill out the questionnaire on the relevant days. However, this did not constitute a problem for the patients, who found it easy to remember because they were doing it on a fixed weekday. Two patients mentioned that a reminder text message would have been advantageous.

#### Questionnaire

The patients reported that the number of items and the length of the questionnaire were appropriate and that reporting on a weekly basis was fitting. A few of the patients would have liked a free text field where they could write a comment or elaborate if the questionnaire did not adequately cover existing symptoms: *“*It is as if you (health care professionals) don’t get enough information” ( woman, 71 years old). The patients were divided when asked if responding to the PROMelanoma questionnaire was reassuring; half of the patients confirmed that this was the case, while the other half rejected this notion: “I feel reassured enough as it is” (woman, 52 years old). The majority reported that their attention to side effects was heightened due to the intervention (eg, “Your focus is increased because you have to remember to write it” [woman, 62 years old]), and that responding to the questionnaire was useful. More of the patients also found that filling out the questionnaire made it easier to remember symptoms when they came to the clinic. One patient reported that she was reminded of her disease every time she responded. A majority of the patients reported that the alert reminding them to contact the department popped up too frequently. As one interviewee put it: “If I were to call every time it pops up, I would have to call very often” (woman, 67 years old). However, if the patients decided that it was not a new symptom or worsening of an already existing symptom, they were able to reject the alert.

#### Patient-Physician Communication

When the patients came to the outpatient clinic, two out of three of the patients who were interviewed felt that the health care professionals had in fact seen their reports and included them in their consultation: “It is like having an agenda for a meeting” (man, 66 years old)*,* “It makes you feel as if you are not just a number in the system” (woman, 49 years old) . A minority did not know if their reports had been seen by the clinician: “I think they have seen it (the report), but it is not something we have discussed” (woman, 69 years old). A few believed that the clinician had in fact not seen it at all, which was of course frustrating due to the fact that they had spent time filling out the questionnaire. One third had the feeling that they contacted the department more as a result of the reporting. Thus, the majority of patients did not think that they were more in touch with the hospital due to the reporting. Overall, the reporting made the patients feel more involved in their treatment and care: “It is nice that we have something common to talk about” (man, 66 years old).

#### Other Themes

Many of the patients explained that a strong motivation for entering the study was that they would be able to help future patients. Of course, they believed that they themselves would benefit, but being able to help others was also important. Including relatives in the reporting was not a theme. The patients did the reporting alone, apart from one patient, and it did not prompt any discussions within the family.

### Focus Group Interview With Clinicians

The participants in the focus group consisted of three doctors and two nurses. They were all women with a median age of 43 years. All of them had broad experience working with cancer patients and dealing with symptoms or side effects (6-11 years). They were also accustomed to caring for melanoma patients receiving immunotherapy. They had all seen the patient reports several times and had included them in the clinician-patient communication.

There was some discrepancy between how the patient and the clinician graded a given symptom. In some cases, the clinician did not find the symptom to be as severe as the patient. In other instances, the clinician felt that the patient had in fact neglected a symptom that they believed should have been reported: “sometimes there’s a discrepancy between what you find out when you talk to the patient and what has been reported … the two things supplement each other” (physician).

Furthermore, the inclusion of patient reporting was seen as being more time-consuming than a typical consultation due to the fact that the clinicians had to log into another system to see the report. Having the reports integrated in the electronic health records (EHRs) was stated not only to save time but also make it much easier to remember to include them in the consultation.

The clinicians agreed that the patients were better prepared when they came to the outpatient clinic, and that the patients had increased focus on their symptoms and were more alert: “I think it is an advantage that the patients become more aware of the side effects that can occur” (nurse). Moreover, the information on toxicity that had been given to the patients prior to treatment start was repeated when the patients responded to the electronic questionnaire at home. Accordingly, there was a better chance that the patients would react appropriately by contacting the department in time instead of waiting for the next scheduled consultation, which might be days or weeks ahead. Thus, having the patients call more often was seen as an advantage because it might enable earlier detection. Moreover, it was an advantage to be able to use the patient reporting as the basis of the consultation by starting with the symptoms that had bothered the patient the most: “…then I scroll down to see where it is red or yellow and that is typically where we start…” (physician). In this way, the patients took part in setting the agenda. However, according to the health care professionals, the patient reporting should be seen as a supplement and not something that could replace the clinician-patient consultation. In addition, the clinicians reported that the eHealth intervention was a valuable tool, particularly for patients who are normally slightly reluctant to contact the department unscheduled: “…it may be precisely the group of patients who are not good at self-care or at least some of them…the weakest patients who…will benefit most from self-reporting by being guided into becoming more aware of when to react to symptoms” (physician). Because the patients were encouraged to make contact if they experienced a new or worsened symptom, they might have felt that it was more legitimate to call the outpatient clinic. All of the clinicians believed that the patients with the best social resources would benefit the least from the intervention because they were sure to contact the department in agreement with the given instructions.

Overall, the clinicians had a positive attitude toward the intervention using an eHealth tool, even though there was also room for improvement in some areas.

### Comparison Between Survey and Patient Interviews With the Focus Group Interview

The clinicians believed that the reporting would make the patients call the hospitals more, whereas the majority of patients did not think that they called more frequently. Some of the patients thought that their reports did not provide the clinicians with enough information; however, none of the clinicians stated this to be the case. Patients and clinicians agreed that the attention to side effects was increased and that the patients were better prepared for the consultation when they came to the outpatient clinic. The patient reports also established a shared agenda for the consultation at the outpatient clinic. Overall, the findings from the survey confirmed what had been established in the patient interviews. The patients reported that it was easy to fill out the questionnaire and that it made sense to do so. Moreover, it increased symptom awareness. Both the patients and clinicians agreed that when the report was in fact included, it helped to prioritize the problems that were most acute.

## Discussion

### Principal Findings

The goal of this study was to elucidate the experiences of malignant melanoma patients and their treating clinicians with an eHealth intervention. Overall, acceptance was high for both clinicians and patients, and both groups believed that it improved communication during the consultation. This is in line with previous studies showing that using PROs prompted patient-clinician dialog, streamlined consultations, and increased focus on side effects [10,42]. In addition, the potential for discrepancies between the degree of severity when clinicians and patients grade a given symptom confirms previous findings [1-4].

However, a minority of the patients in this study did not believe that the clinician had actually seen their reports when they came to the clinic. This point was primarily expressed by patients who were enrolled at the beginning of the study, when monitoring the patient reports had not yet become routine in the outpatient clinic. This improved over time as the clinicians got used to taking the reports into consideration. This finding is in line with Mooney et al [43], who argued that when the advantages of systematic PRO collection in clinical care become visible, adoption will rapidly occur. Although the use of PRO in the clinic can improve communication, it does not necessarily result in intensified symptom treatment and improved symptom management [44]. Thus, it remains to be seen if patient and clinician satisfaction with the eHealth intervention will translate into a reduction in symptom severity; this aspect is being investigated in the ongoing RCT PROMelanoma.

As for the survey, patient satisfaction was extremely high for many of the questions. The three items that had the lowest scores in satisfaction (items 8, 9, and 10) deal with the inclusion of patient response in the clinic. This response is comparable with the results of other studies using the Patient Feedback Form [19]. This suggests that one of the challenges when using PROs may be to ensure that the patients’ responses from questionnaires are included in treatment and care. For many years, PROs have been collected in clinical trials, but they have not been used routinely in clinics. It will likely take some time before implementing PROs in clinical practice becomes as natural as other procedures within the health care system.

The clinicians participating in the focus group interview agreed that the least resourceful patients would benefit most from the eHealth intervention, because they were usually less inclined to contact the clinic in case of any symptoms. This notion has been confirmed in other studies, which have shown that the level of patient involvement is dependent on the degree of health literacy. For example, patients with a high level of education are more inclined to be involved in medical decision making compared to patients with a low level of education [45]. Basch et al [46] also suggested that patients who do not have any computer experience may have weaker communication skills and therefore benefit more from a structured setup. It can be argued that if this patient group becomes involved in the reporting of side effects, they may be encouraged to react appropriately when an alert is triggered, thereby potentially improving toxicity management. When data from the RCT on the number of phone contacts are analyzed, it will be revealed if patients in the intervention arm actually did call more frequently. Preliminary findings revealed that 78% of the patients adhered to the intervention by reporting their symptoms on a weekly basis.

Some of the patients also argued that the eHealth intervention was very box-like and they would have liked a space where they could write more about their symptoms instead of just checking a box. The patients in the PROMelanoma study can add other symptoms as advised by the NCI, but the patients also wished to be able to elaborate on some of the symptoms. Although this is understandable from a patient point of view, one must keep in mind that the primary aim of introducing the intervention was to increase patient awareness, hoping to reduce the number of severe side effects and improve clinical outcome. Further, it was important that it was fairly easy and not too time-consuming for the clinicians to acquire a quick outline of the reporting if it were to be implementable in the clinic. Moreover, patients had the opportunity to elaborate on the various symptoms that they experienced when they came to the clinic.

### Limitations

One potential limitation of the study is that a deductive approach was used by having the coding framework decided in advance, which may limit the development of new themes [28]. By using a deductive approach, and thus imposing our own structure on the data, the analysis may have been biased. However, the fact that we had some knowledge about the subject made the deductive approach an obvious choice. The fact that one of the predetermined categories, involvement of relatives, did not develop into a theme and was removed during the analysis indicates that we were not too locked in our preconception.

An obvious limitation is that we were only able to conduct one focus group interview with the clinicians. However, we aimed at selecting participants with vast knowledge and expertise of the subject [47], which limited the potential number of informants. Of course, other physicians and nurses had treated these patients, but the fact that they did not do so on a routine basis made them unsuitable as participants. Consequently, we settled for one focus group although it would have been preferable to have more. With respect to the number of interviewed patients, we judged that data saturation was reached at patient number 14, as data were replicated, which is why we stopped including patients in the study at this point. According to Francis et al [48], data saturation may very well be reached after 14 interviews when diversity sampling is appropriate. We believed this was the case in this study.

Another potential limitation is that the alert function was triggered too frequently according to the majority of patients. This may be changed when designing future studies or implementing the intervention beyond the study period to avoid alert fatigue. Nevertheless, having an alert function is a good idea, as studies have shown that patients value advice on when it is appropriate to contact the hospital [49]. In addition, it is vital that the clinicians log into the system and see the patients’ reports prior to every consultation. Otherwise, patients may lose the incentive to continue to fill out the reports. PROs must be implemented in such a way that the process is embedded as part of routine care [21] so that clinicians do not have to be reminded to view the patient report (eg, by project managers or study coordinators). In this regard, it is important that PROs are easily accessible to clinicians (ie, integrated in the EHR) to be successful, as recommended by the clinicians in the focus group interview. Recommendations on how to integrate PROs into the EHR have already been developed by the PRO-EHR Users’ Guide Steering and Working Groups [50].

### Conclusion

We found a high acceptance of the eHealth intervention tool among clinicians and melanoma patients being treated with immunotherapy. The tool was easy to use and contributed to greater symptom awareness and patient involvement. Thus, in terms of patient and clinician satisfaction, it makes sense to continue using the tool beyond the project period. However, it remains to be investigated whether the predominantly positive perceptions of the intervention by patients and clinicians will also be followed by a reduction in the number of severe side effects. Our RCT PROMelanoma will shed light on this aspect.

## Abbreviations

COREQ: Consolidated Criteria for Reporting Qualitative Research
CTCAE: Common Terminology Criteria for Adverse Events
EHR: electronic health record
ePROs: electronic patient reported outcomes
NCI: National Cancer Institute
PROs: patient reported outcomes
RCT: randomized controlled trial
